# Khellin and Visnagin Differentially Modulate AHR Signaling and Downstream CYP1A Activity in Human Liver Cells

**DOI:** 10.1371/journal.pone.0074917

**Published:** 2013-09-19

**Authors:** Radim Vrzal, Katrin Frauenstein, Peter Proksch, Josef Abel, Zdenek Dvorak, Thomas Haarmann-Stemmann

**Affiliations:** 1 Department of Cell Biology and Genetics, Faculty of Science, Palacky University, Olomouc, Czech Republic; 2 IUF - Leibniz Research Institute for Environmental Medicine, Düsseldorf, Germany; 3 Institute of Pharmaceutical Biology and Biotechnology, Heinrich-Heine-University, Düsseldorf, Germany; Florida International University, United States of America

## Abstract

Khellin and visnagin are two furanochromones that can be frequently found in ethnomedical formulations in Asia and the Middle East. Both compounds possess anti-inflammatory and analgesic properties, therefore modern medicine uses these compounds or structurally related derivatives for treatment of vitiligo, bronchial asthma and renal colics. Despite their frequent usage, the potential toxic properties of visnagin and khellin are not well characterized up-to-now. Many natural compounds modulate the expression and activity of cytochrome P450 1A1 (CYP1A1), which is well-known to bioactivate pro-carcinogens. The expression of this enzyme is controlled by the aryl hydrocarbon receptor (AHR), a ligand-activated transcription factor and regulator of drug metabolism. Here, we investigated the influence of both furanochromones on AHR signaling in human HepG2 hepatocarcinoma cells and primary human hepatocytes. Both compounds transactivated xenobiotic response element (XRE)-driven reporter gene activity in a dose-dependent manner and induced CYP1A1 transcription in HepG2 cells and primary hepatocytes. The latter was abolished in presence of a specific AHR antagonist. CYP1A enzyme activity assays done in HepG2 cells and primary hepatocytes revealed an inhibition of enzyme activity by both furanochromones, which may become relevant regarding the metabolism of xenobiotics and co-administered therapeutic drugs. The observed induction of several other members of the AHR gene battery, whose gene products are involved in regulation of cell growth, differentiation and migration, indicates that a further toxicological characterization of visnagin and khelllin is urgently required in order to minimize potential drug-drug interactions and other toxic side-effects that may occur during therapeutic usage of these furanochromones.

## Introduction

The aryl hydrocarbon receptor (AHR) is a ligand-dependent transcription factor that is activated by dioxins, polycyclic aromatic hydrocarbons (PAHs) and related environmental pollutants [1,2]. Gene disruption studies in mice have identified the AHR as a crucial mediator of PAH carcinogenicity [3] and dioxin toxicity, including immune- and hepatotoxic effects [4,5]. In the absence of a ligand, the AHR is trapped in a cytosolic multiprotein complex consisting of heat shock protein 90, AHR interacting protein, and co-chaperone p23 [2]. In addition, an association with the soluble tyrosine kinase c-src is discussed [6]. Upon ligand-binding, this complex dissociates and the AHR shuttles into the nucleus, dimerizes with its partner molecule AHR nuclear translocator (ARNT) and binds to xenobiotic responsive elements (XRE) in the promoter region of target genes to stimulate their expression [2]. The AHR gene battery encodes for drug metabolizing enzymes as well as for proteins involved in cell growth and differentiation. The probably best examined target molecules of AHR signaling are cytochrome P450 (CYP) family 1 enzymes, which are involved in the oxidative metabolism of PAHs and other polyaromates, including steroid hormones and therapeutic drugs [7]. Beside direct induction of XRE-dependent gene expression, the AHR-driven activation of the c-src kinase initiates an alternative route of AHR signaling, sequentially comprising the phosphorylation of the EGF receptor, stimulation of downstream MAPKs and transcriptional induction of another set of target genes [8].

Several studies provided evidence that the AHR is not only activated by anthropogenic chemicals, but also by natural and endogenous ligands [1,2]. For instance, 6-formylindolo[3,2b] carbazole, a tryptophan photoproduct, which is intracellularly formed upon ultraviolet (UV) B irradiation, was identified as a potent AHR agonist and crucial mediator of the UVB response in human keratinocytes [9]. In addition, numerous plant polyphenols and alkaloids were identified to stimulate or repress AHR signaling and downstream CYP1 enzyme activity [1,2]. Besides influencing the metabolic activation of PAHs, aflatoxins, and related procarcinogens, the modulation of CYP1 activity by food constituents, herbal remedies or lifestyle-derived factors can directly affect the metabolic fate and therapeutic efficiency of co-administered medications. For instance, exposure of rats to the strong AHR agonist and CYP1A inducer rutaecarpine [10] was shown to significantly alter the pharmacokinetics of drugs, such as acetaminophen and theophylline [11,12].

Khellin and the structurally related furanochromone visnagin are the major active principles found in *Ammi* *visnaga* , a widespread flowering plant whose dried fruits have been traditionally used in Asia and the Middle East for treatment of coronary diseases, bronchial asthma, renal colics and muscle spasms [13,14]. Both compounds exhibit vasodilatory activities due to their calcium channel blocking properties [15,16] and act in an anti-inflammatory manner by inhibiting AP-1 and NF-κB signaling [17]. Khellin has been extensively used by the pharmaceutical industry as basic raw material for the development of drugs such as the anti-asthmatic agent sodium cromoglycate or the widely used anti-arrhythmic drug amiodarone [18]. Recent studies identified derivatives of khellin and visnagin as suitable agents to treat different types of tumors, epileptic seizures, kidney stones and inflammatory diseases [19–22]. Due to its photosensitizing properties and lesser phototoxic side-effects compared to psoralens, khellin is also used in the photochemotherapy (khellin treatment plus ultraviolet A irradiation; KUVA) of vitiligo, a pigmentation disorder of the skin [23,24].

Because (1) khellin and visnagin probably fulfill the structural prerequisites to bind to the AHR [25], (2) structurally related furocoumarins (8-methoxypsoralen, angelicin) are known to stimulate AHR-dependent CYP1A1 expression in rat hepatocytes [26], and (3) UVB-induced skin pigmentation is partially mediated by AHR [27,28], we here investigated if the two furanochromones activate AHR signaling in human primary hepatocytes and hepatocarcinoma cells.

## Materials and Methods

### Ethics Statement

Our laboratory obtained the approval from ethics committee (The Ethics Committee of University Hospital, Olomouc and the Faculty of Medicine Palacky University in Olomouc), reference number 119/07 for handling the liver tissue for hepatocytes isolation. The liver tissue samples were procured from The Centre of Transplantation (http://www.fnol.cz/kliniky-ustavy-oddeleni.asp#seznam). We are associated with the University Hospital and the Faculty of Medicine Palacky University in Olomouc.

### Chemicals

Visnagin (purity: 97%) was purchased from Acros Organics (Geel, Belgium), khellin (purity: 98%) and 3-methylcholanthrene (3MC) from Sigma-Aldrich (Munich, Germany). 2,3,7,8-tetrachlorodibenzo-p-dioxin (TCDD) was purchased from Ultra Scientific (Kingstown, RI, USA). MNF was kindly donated by Gabriele Vielhaber (Symrise GmbH & Co. KG, Holzminden, Germany). All other chemicals were of the highest quality commercially available.

### Cell Culture

#### a) Primary Human Hepatocytes

Human liver tissue used in this study was obtained from two sources: (i) from multiorgan donors LH40 (male; 57 years), LH42 (female; 60 years); tissue acquisition protocol was in accordance with the requirements issued by local ethical commission in the Czech Republic; (ii) long-term human hepatocytes in monolayer Batch HEP220586 (male; 82 years), HEP220624 (male; 80 years) (Biopredic International, Rennes, France). The research was not conducted outside of my country of residence. Cells were cultured in serum-free medium. Hepatocytes were treated for 24 h or 48 h with visnagin or khellin, 3MC (1 µM) or TCDD (5 nM) and/or vehicle (DMSO; 0.1% v/v). Cultures were maintained at 37 °C and 5% CO_2_ in a humidified incubator.

#### b) HepG2 Cells

Human Caucasian hepatocellular carcinoma cells HepG2 (ECACC No. 85011430) were cultured in Dulbecco’s modified Eagle’s medium (DMEM) supplemented with 10% of fetal calf serum, 100 U/mL streptomycin, 100 µg/mL penicillin, 4 mM L-glutamine, 1% non-essential amino acids, and 1 mM sodium pyruvate. Cells were maintained at 37°C and 5% CO_2_ in a humidified incubator.

### Reporter Gene Assay

Experiments were performed in stably transfected gene reporter cell line AZ-AHR, which was derived from HepG2 cells transfected with a construct containing several XRE 5’-upstream of luciferase reporter gene [29]. Following the plating, cells were stabilized for 16 h and then treated for 24 h with visnagin/khellin (0.001-20 µM), 3MC (5 µM) and/or vehicle (DMSO; 0.1% v/v). After treatment, cells were lysed and luciferase activity was measured using an Infinite M200 machine (Tecan, Grödig, Austria).

### RNA Isolation, Reverse Transcription and PCR

#### a) Primary Human Hepatocytes

Total RNA was isolated from primary hepatocytes in the laboratory of R.V and Z.D. using *TRI Reagent* **®** (Molecular Research Center, Cincinnati, OH, USA). cDNA was synthesized from 1000 ng of total RNA using M-MLV Reverse Transcriptase (Finnzymes, Espoo, Finland) at 42 °C for 60 min in the presence of random hexamers (Takara, Shiga, Japan). qRT-PCR was carried out using LightCycler FastStart DNA Master^PLUS^ SYBR Green I (Roche Diagnostic Corporation, Prague, Czech Republic) on a Light Cycler 480 II apparatus (Roche Diagnostic Corporation). CYP1A1 and GAPDH mRNA expression was determined as described previously [30]. Measurements were performed in triplicates. Gene expression was normalized to GAPDH as a housekeeping gene.

#### b) HepG2 Cells

Total RNA was isolated from HepG2 cells in the laboratory of T.H.S. using the peqGOLD total RNA kit (Peqlab, Erlangen, Germany). For each sample 0.5µg of total RNA was reverse transcribed using MMLV reverse transcriptase (Promega, Madison, WI) in a total volume of 20µl. 3µl of cDNA of a 1:3 dilution were used for qRT-PCR in a Corbett-Rotor Gene 300 light cycler (Qiagen, Hilden, Germany) with QuantiFast SYBR Green (Qiagen). All samples were measured in triplicate. Gene expression was normalized to β-actin as a housekeeping gene. The oligonucleotides for amplification were described previously: β-actin, CYP1A1, CYP1B1, and AHRR [31], plasminogen activator inhibitor-2 (PAI-2) [32], and vascular endothelial growth factor (VEGF) [33].

### SDS-PAGE and Western Blot Analysis

Total protein extracts for each sample were prepared from 1 well of 6-well plate dish. Cells were washed twice with ice-cold PBS and scraped into 1 ml of PBS. The suspension was centrifuged (2,300x g/2 min/4°C) and the pellet was re-suspended in 150 µl of ice-cold lysis buffer (150 mM NaCl; 10 mM Tris pH 7.2; 0.1% (w/v) SDS; anti-protease cocktail, 1% (v/v) Triton X-100; anti-phosphatase cocktail, 1% (v/v) sodium deoxycholate; 5 mM EDTA). The mixture was vortexed and incubated for 10 min on ice and then centrifuged (15,700x g/13 min/4°C). Supernatant was collected and the protein content was determined by the Bradford reagent. SDS–PAGE gels (8%) were run on a BioRad apparatus according to the general procedure followed by the protein transfer onto PVDF membrane. The membrane was saturated with 5% non-fat dried milk for 1 h at room temperature. Blots were probed with primary antibodies against CYP1A1 (goat polyclonal, sc-9828, G-18, diluted 1:500 – for detection in human hepatocytes; rabbit polyclonal, sc-20772, H-70, diluted 1:500 – for detection in HepG2 cells), CYP1B1 (mouse monoclonal, sc-374228, G-4, 1:1000), actin (goat polyclonal; sc-1616, 1-19, diluted 1:2000), all purchased from Santa Cruz Biotechnology (Santa Cruz, CA, USA) or GAPDH (rabbit monoclonal, 2118, 14C10, diluted 1:1000) purchased from Cell Signaling Technology, overnight at 4°C. Chemiluminescence detection was performed using horseradish peroxidase-conjugated secondary antibodies (Santa Cruz Biotechnology) and Western blotting Luminol kit (Santa Cruz Biotechnology). Densitometric analyses were carried out using the AlphaEaseFC Software (Alpha Innotech, San Leandro, CA).

### CYP1A Enzyme Activity Assay

7-Ethoxyresorufin-O-deethylation (EROD) activity was determined in intact HepG2 cells or hepatocytes in 96-well plate format. The cellular monolayers were washed twice with PBS and then they were incubated with 100 µl of the PBS containing 8 µM 7-ethoxyresorufin and 10 µM dicumarol to prevent the further metabolism of resorufin. After 30 min of incubation at 37°C, 75 µl was transferred to black 96-well plate together with 125 µl of methanol. The fluorescence of resorufin was measured at 530 nm excitation and 590 nm emission wavelengths using an Infinite M200 machine (Tecan). The results were normalized on cell viability determined by MTT assay in order to exclude cytotoxicity.

### Statistical Analyses

Results were expressed as mean ± standard deviation. Paired Student’s t-test was applied to all analyses, *p* values ≤ 0.05 were considered as significant.

## Results and Discussion

In this study, we asked if an exposure of human liver cells to khellin and the closely related compound visnagin has an impact on the activation of the AHR and its downstream targets. Although, both furanochromones are often used in alternative medicine, especially the potential toxic effects provoked by khellin are of interest, since it is frequently used for photochemotherapy of cutaneous pigmentation disorders.

**Figure 1:**
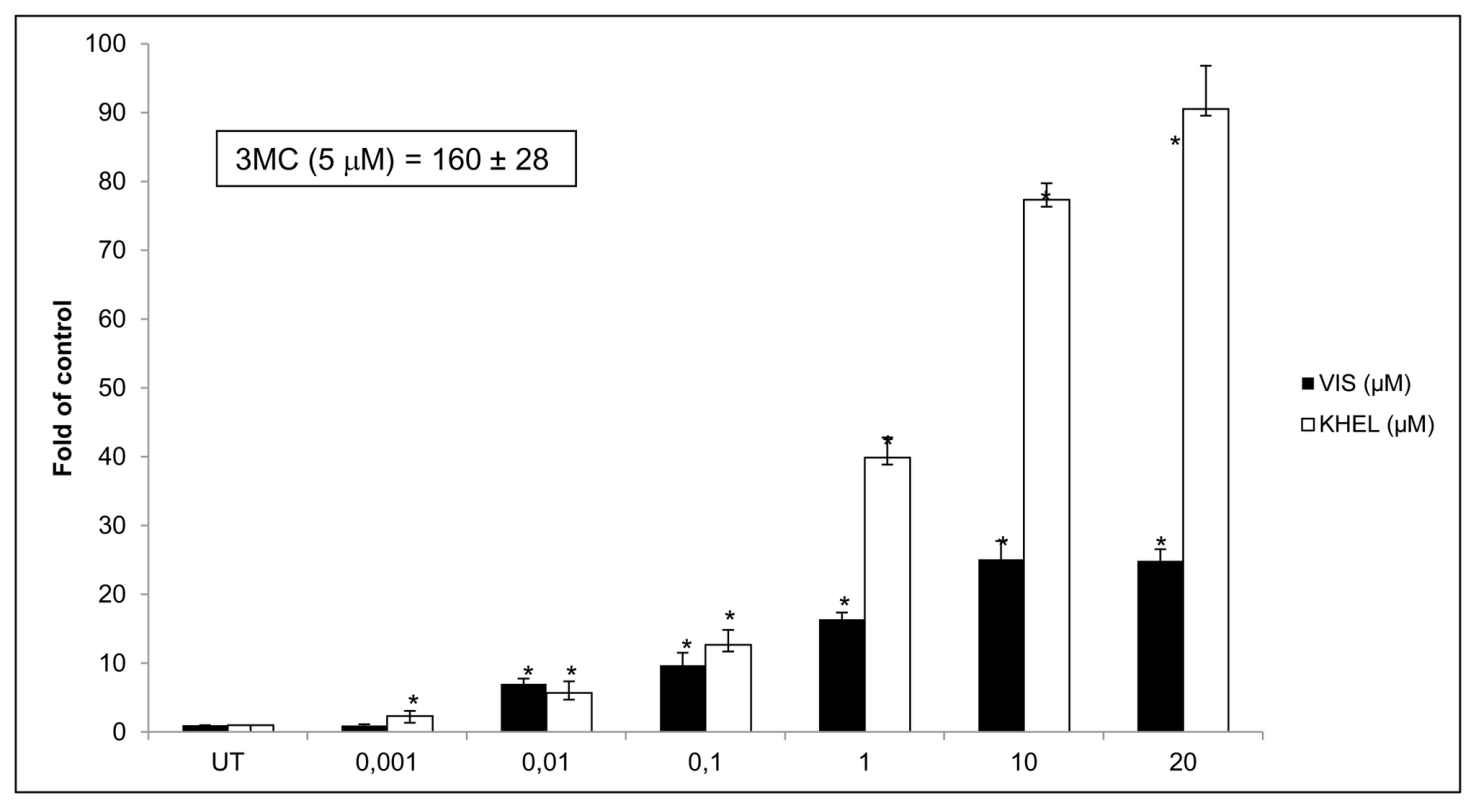
Visnagin and khellin stimulate XRE-driven luciferase activity in AZ-AHR cells. Stably transfected AZ-AHR cells were treated for 24 h with visnagin (VIS; 0.001 µM-20 µM), khellin (KHEL, 0.001 µM-20 µM), 5 µM 3MC and/or vehicle (DMSO; 0.1% v/v). Analyses were performed in four independent experiments and are expressed as fold induction over untreated cells. * - Value is significantly different from DMSO-treated cells (*p* < 0.05).

**Figure 2:**
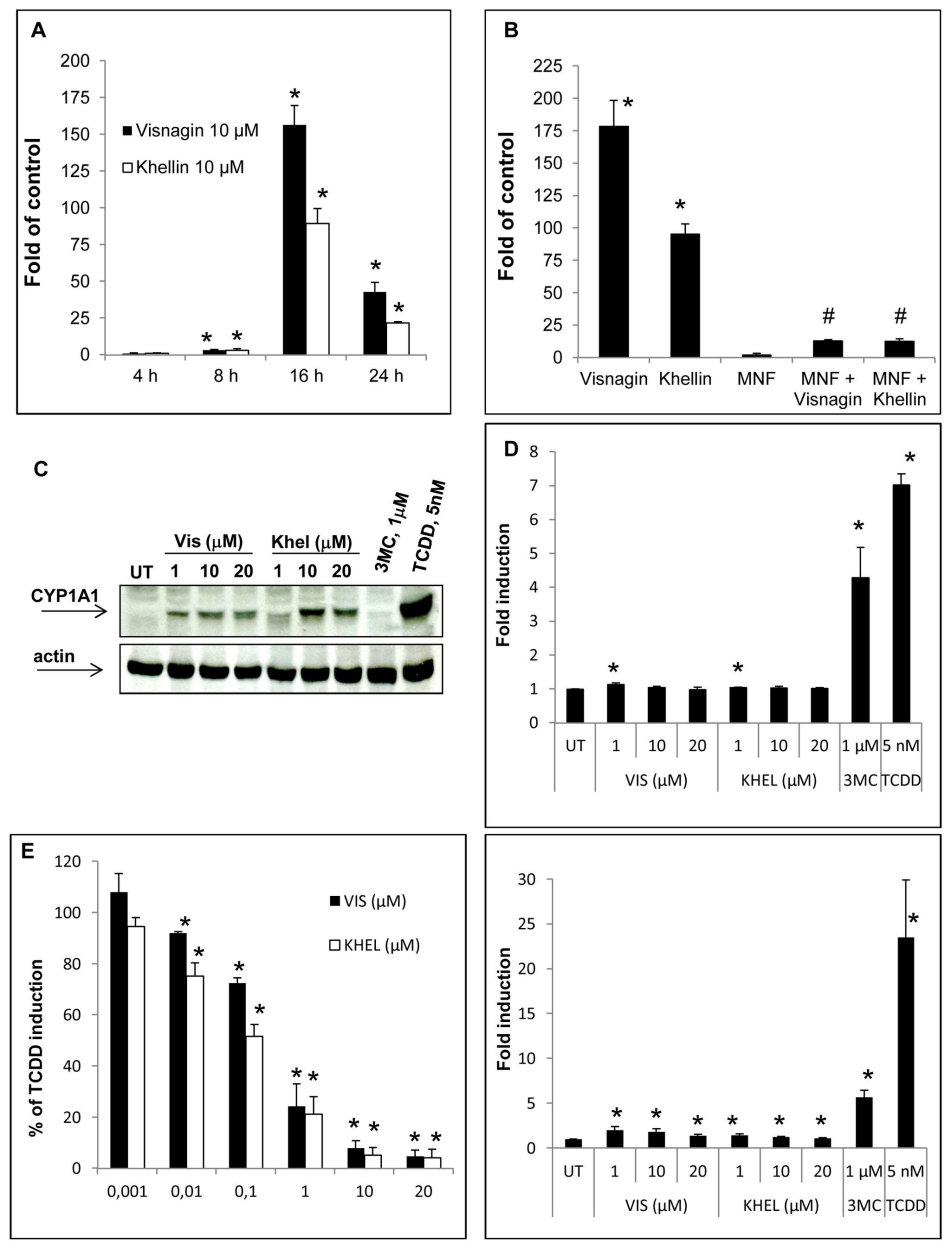
Effect of visnagin and khellin exposure on CYP1A1 expression and activity in HepG2 cells. A) HepG2 cells were treated with 10 µM visnagin, 10 µM khellin, and/or DMSO for 4 h, 8 h, 16 h, and 24 h. The results from PCR are shown as fold of DMSO-treated control cells. The data are mean from three independent experiments and were normalized to beta-actin transcription. * - value is significantly different from DMSO-treated cells (*p* < 0.05). B) HepG2 cells were pre-treated with 20 µM MNF for 1 h and then exposed to 10 µM visnagin or 10 µM khellin for additional 16 h. The results from PCR are shown as fold of DMSO-treated control cells. The data are mean from three independent experiments and were normalized to beta-actin transcription. * - value is significantly different from DMSO-treated cells (*p* < 0.05). # - value is significantly reduced in comparison to cells treated with VIS and KHEL, respectively; (*p* < 0.05). C) HepG2 cells were treated with visnagin (VIS; 1 µM-20 µM), khellin (KHEL; 1 µM-20 µM), 1 µM 3MC, 5 nM TCDD, and/or vehicle (DMSO; 0.1% v/v) for 48 h. Thereafter, western blotting analyses for detection of CYP1A1 and actin were performed as described in Materials and Methods section. The representative western blot analysis of two independent experiments (passages) is presented. D) HepG2 cells were treated with visnagin (VIS; 1 µM-20 µM), khellin (KHEL; 1 µM-20 µM), 1 µM 3MC, 5 nM TCDD, and/or vehicle (DMSO; 0.1% v/v) for either 16 h (upper panel) or 48 h (lower panel). EROD activity was determined as described in Materials and Method section. Analyses were performed in three independent experiments and are shown as fold induction over untreated cells. * - value is significantly different from DMSO-treated cells (*p* < 0.05). E) HepG2 cells were treated with TCDD (5 nM) for 48 h. Thereafter, substrate mixture was supplemented with increasing doses of visnagin (VIS 1 nM -20 µM) or khellin (KHEL; 1 nM -20 µM) and EROD activity was determined as described in Materials and Methods section. Data are mean from three independent experiments and are expressed as percentage (%) of TCDD-mediated induction (i.e. induction by TCDD = 100%). * - value is significantly different from TCDD-treated cells (*p* < 0.005).

A 24 h treatment of AZ-AHR reporter cells, a HepG2 cell line harboring a stably transfected XRE-driven reporter gene construct [29], with increasing concentrations of khellin and visnagin (0.001 µM to 20 µM) resulted in a dose-dependent increase of reporter gene activity (Figure 1). A maximum 24-fold (for visnagin) and 83-fold (for khellin) induction rate was observed in cells treated with 20 µM of the respective test compound. Noteworthy, a first statistical significant increase in luciferase activity was already observed after administration of 1 nM khellin and 10nM visnagin, respectively. Treatment of the AZ-AHR cells with 5 µM of the potent AHR agonist 3MC (positive control) led to a higher, roughly 160-fold induction rate of XRE-driven promoter activity (Figure 1). These data point to the idea that both furanochromones are moderate activators of hepatic AHR signaling. To further confirm this notion, we analyzed the time-dependent effect of khellin (10 µM) and visnagin (10 µM) exposure on mRNA expression of CYP1A1 in HepG2 cells. As shown in Figure 2A, a slight induction of CYP1A1 expression was observed after 8 h of treatment, whereas the peak expression was reached additional 8 h later. At this time point, visnagin caused an approximately 160-fold induction of CYP1A1 transcription, whereas khellin enhanced the expression rate roughly 90-fold. This was surprising, since in the reporter gene assays, khellin turned out to be the more potent activator of the XRE-driven reporter gene construct. Noteworthy, we and others have earlier described similar discrepancies between the results obtained from XRE-driven reporter gene assays and other indicator tests for AHR activation, such as gel retardation assays [34], EROD assays [10], and gene expression analyses [35].

**Figure 3:**
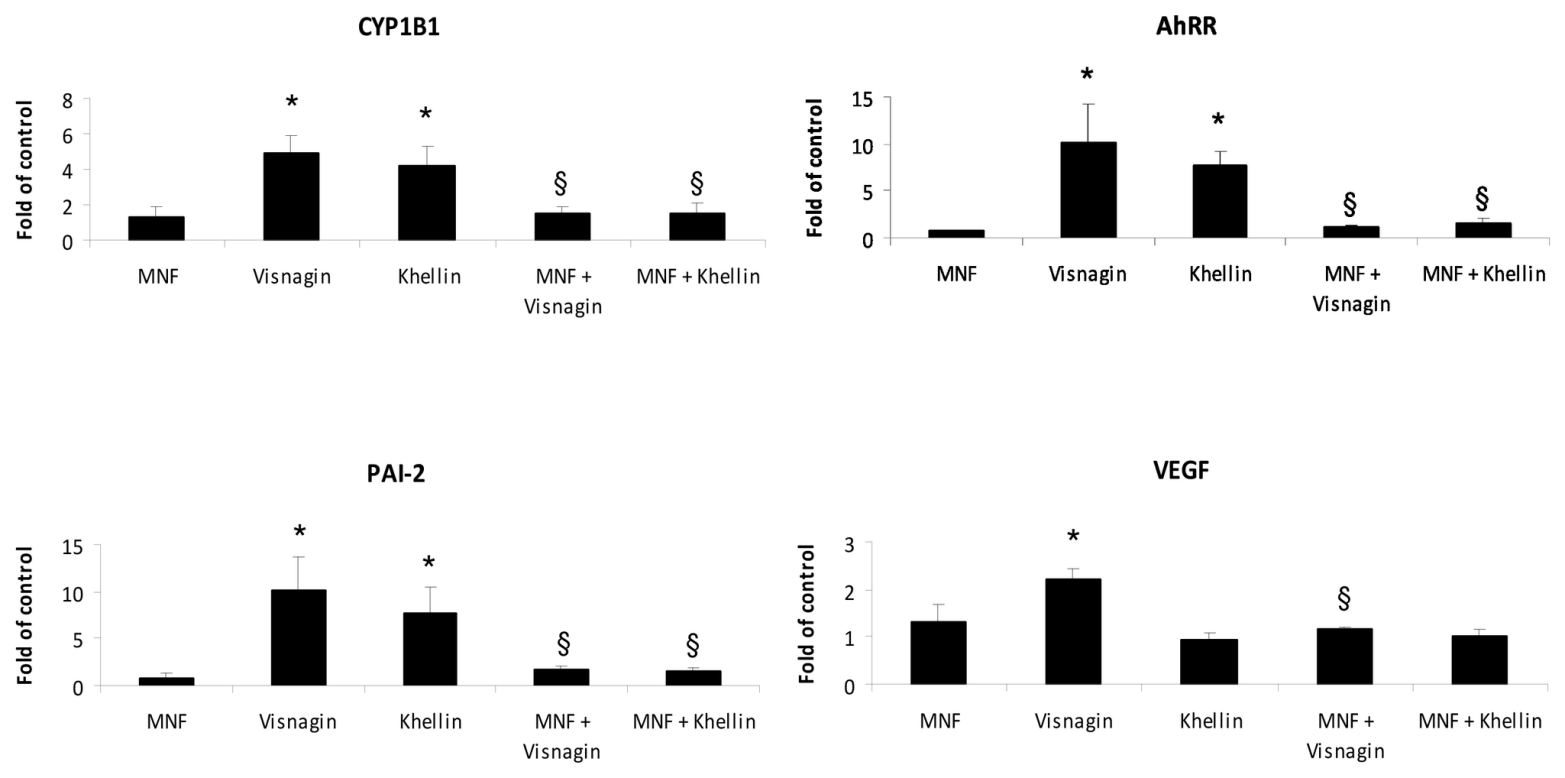
Visnagin and khellin modulate the expression of several AHR target genes in HepG2 cells. HepG2 cells were pre-treated for 1 h with 20 µM MNF or 0.1% (v/v) DMSO and were subsequently exposed to 10 µM visnagin (VIS) or 10 µM khellin (KHEL). After 16 h, RNA was isolated and reverse transcribed and the expression of CYP1B1, AhRR, PAI-2 and VEGF was measured by qRT-PCR. The data are mean from three independent experiments and were normalized to beta-actin expression. * - value is significantly increased compared to DMSO-treated cells; § - value is significantly reduced in comparison to cells treated with VIS and KHEL, respectively; (*p* < 0.05).

To prove a direct involvement of the AHR in the observed transcriptional changes, we introduced the specific AHR antagonist 3’-methoxy-4’-nitroflavone (MNF) [36] to our expression analyses (Figure 2B). For this purpose, HepG2 cells were pre-treated for 1 h with MNF (or solvent) and subsequently were co-exposed for 16 h to 10 µM of either visnagin or khellin. Whereas MNF exposure alone did not significantly alter basal CYP1A1 expression, the visnagin- and khellin-mediated induction of CYP1A1 mRNA was clearly abolished in the co-exposure scenario (Figure 2B). Therefore, it is highly likely that the two furanochromones bind to the AHR and modulate downstream gene expression. Since the induction of mRNA is often correlated with the induction of protein, we performed western blotting analysis for CYP1A1. As a positive control we used 1 µM 3MC and 5 nM TCDD. We observed massive induction of CYP1A1 protein by TCDD but almost unimportant induction by 1 µM 3MC after 48 hrs (Figure 2C). However, both furanochromones induced CYP1A1 protein to level exceeding DMSO- as well as 3MC-treated cells (Figure 2C). To test if the enhanced CYP1A1 gene expression was translated into corresponding enzyme activities, we assayed the CYP1A1/1A2-mediated 7-ethoxyresorufin-O-dealkylase (EROD) activity in HepG2 cells treated with increasing concentrations of visnagin or khellin (Figure 2D). Treatment of the cells for 16 h and 48 h with 1 µM 3MC resulted in a 4.3- and 5.7-fold increase of EROD activity. Exposure to 5 nM TCDD increased CYP1A enzyme activity 7- (16 h) and 23.5-fold (48 h), respectively. In contrast to these high-affinity AHR ligands, neither khellin nor visnagin treatment led to a significant induction of CYP1A enzyme activity after 16 h. After 48 h, we observed a slight but significant enhancement of EROD activity, which, at least for visnagin, displayed a reverse dose-response, pointing to a possible inhibition of CYP1A enzyme activities. To verify this, we pretreated HepG2 cells with 5 nM TCDD for 48 h and consequently treated the cells with the substrate mixture containing 7-ethoxy-*O*-resorufin with or without visnagin or khellin. As shown in Figure 2E, the TCDD-induced catalytic activity was decreased in a dose-dependent manner by both compounds. Even though this observation may theoretically also reflect an interference of visnagin/khellin with the cellular uptake of 7-ethoxy-*O*-resorufin, the most likely explanation is a visnagin/khellin-mediated inhibition of CYP1A catalytic activity. This finding is in accordance with earlier studies on S9-treated *Salmonella* *typhimurium* TA98 cultures, showing that khellin exposure reduced the metabolic activation of various pro-mutagenic PAHs [37] and 2-amino-3-methylimidazo(4,5-f)-quinoline, which is mainly activated via CYP1A-mediated N-hydroxylation [38]. In combination with our results, exposure to visnagin and khellin would probably rather inhibit metabolic activation of pro-carcinogens than inducing it. The other way round, the inhibition of CYP1A enzyme activities may lead to alterations in the pharmacokinetics of drugs, such as the leukotriene receptor antagonist verlukast [39], the antipsychotic drug clozapine [40], and the calcium channel blocker verapamil [41]. In addition, visnagin- and khellin administration did not only modulate CYP1A1 expression but also modulated the transcription of CYP1B1 in an AHR-dependent manner. As shown in Figure 3, visnagin and khellin treatment elevated CYP1B1 gene expression, whereas MNF successfully counteracted this induction. CYP1B1 is involved in steroid breakdown [42] and of crucial relevance regarding the carcinogenicity of certain PAHs, especially 7,12-dimethylbenz(a) anthracene [43,44]. Moreover, we observed increased level of CYP1B1 protein in the presence of both furanochromones as well (Figure S1).

Beside their effect on the expression of the prototype target genes CYP1A1 and CYP1B1, we investigated if khellin and visnagin can influence the transcription of other AHR target genes (Figure 3). To this aim we pretreated HepG2 cells for 1 h with 20 µM MNF (or solvent) and subsequently exposed them for 16 h to 10 µM visnagin or 10 µM khellin, respectively. Likewise CYP1, gene expression of the AHRR is also regulated via functional XRE located in its enhancer/promoter sequence [45,46]. As expected, treatment of HepG2 cells with visnagin and khellin resulted in an increased expression of AHRR, which was also significantly attenuated by MNF pre-treatment. The AHRR protein is a negative feedback inhibitor of AHR signaling that also dimerizes with ARNT and, due to the lack of a C-terminal transactivation domain, terminates XRE-dependent transcription [47]. Recently, a study on primary human mammary epithelial cells and human lung cancer cells provided evidence that the AHRR is not just a negative regulator of AHR, but also a critical regulator of cell growth and apoptosis [48]. PAI-2 is an important factor influencing the growth and differentiation of cells by regulating proteolysis of the extracellular matrix [49], and PAI-2 was previously shown to be up-regulated in an AHR-dependent manner [50]. VEGF expression is induced during hypoxia to promote proliferation and migration of endothelial cells and thus is an important trigger for vasculogenesis and angiogenesis during embryonic development, wound healing and tumor growth [51,52]. Exposure of MCF10A cells to dioxin was shown to result in an increased expression of PAI-2 and VEGF, which was blunted in presence of either MNF or PP2, a src kinase inhibitor, indicating that both genes are regulated through the c-src-dependent, non-genomic AHR signaling pathway [53]. As shown in Figure 3, treatment of HepG2 cells with 10 µM visnagin or khellin also enhanced PAI-2 transcription in an AHR-dependent manner, as indicated by the samples co-treated with MNF. However, only visnagin significantly induced VEGF transcription in the tested concentration. This induction was again blocked by MNF co-exposure. These results strongly indicate that the two furanochromones stimulate both XRE-dependent as well as XRE-independent, non-genomic AHR signaling. This in turn points to the idea that visnagin and khellin may affect cellular functions and pathways beyond CYP-mediated metabolism, and thus may contribute to pathophysiological processes in human hepatic cells.

**Figure 4:**
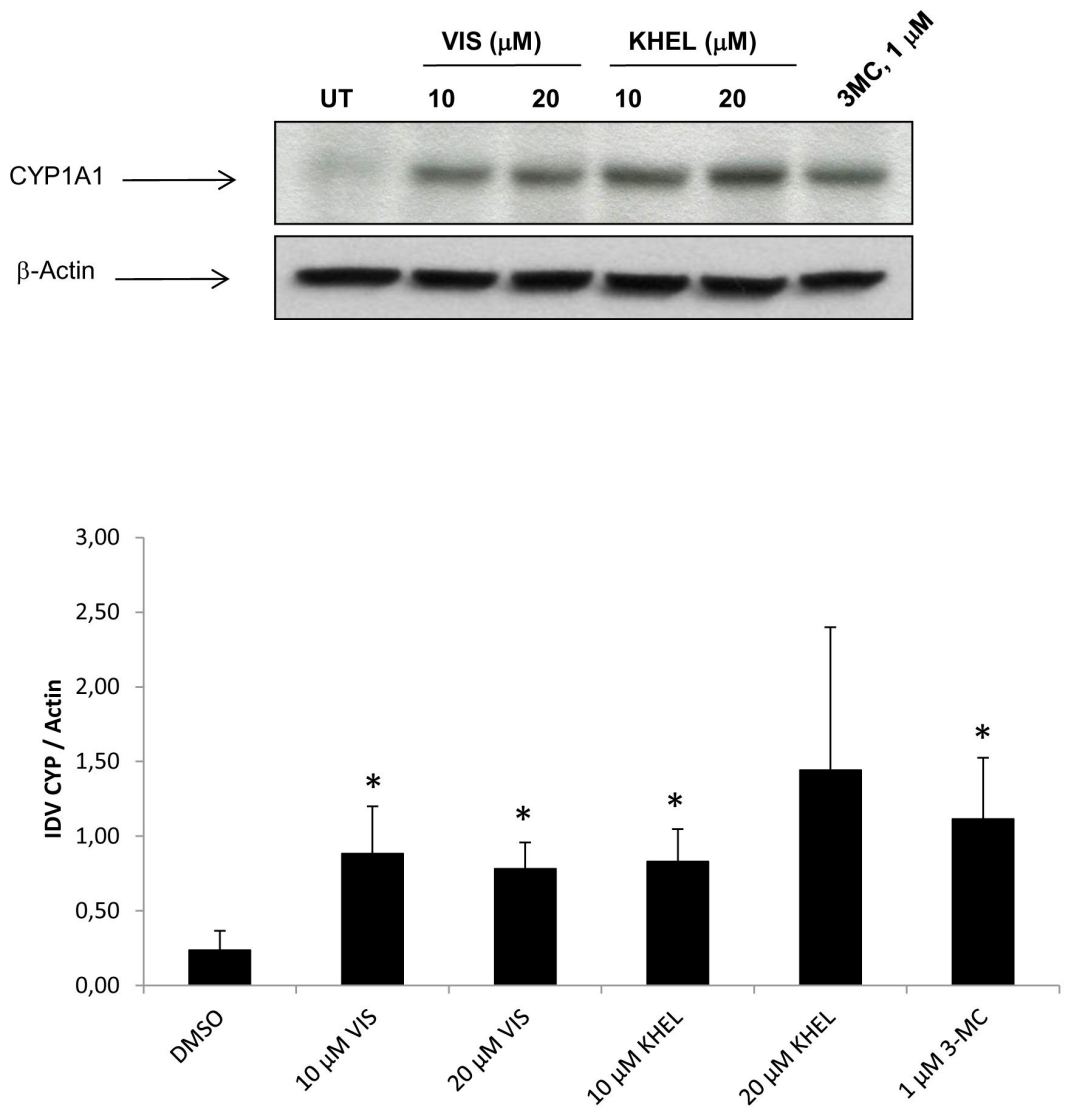
Effect of visnagin and khellin exposure on CYP1A1 protein expression in human primary hepatocytes. Human hepatocytes were treated with visnagin (VIS; 10 µM and 20 µM), khellin (KHEL; 10 µM and 20 µM), 1 µM 3MC, and/or vehicle (DMSO; 0.1% v/v) for 48 h. The shown western blot panels were obtained using protein lysate from the primary hepatocyte culture LH40. The densitometric analysis presents the results from western blot experiments performed with protein lysates form four different hepatocyte donors (LH42, HEP220624, HEP220586, and LH40; IDV = integrated density volume); (*p* < 0.05).

To ensure that the AHR-activating properties identified so far were not restricted to the used hepatocarcinoma cell line, we exposed primary human hepatocytes for 24 h to visnagin and khellin and subsequently investigated CYP1A1 mRNA as well as protein expression. As expected, the AHR agonists used as positive controls, 3MC (1 µM) and TCDD (5 nM), significantly induced CYP1A1 mRNA expression (Table 1). The induction varied greatly among the hepatocyte cultures from different donors, pointing to the presence of interindividual differences in CYP1A1 responsiveness, probably due to age- and gender-related factors [54]. However, this was probably not our case since when we compared basal CYP1A1 mRNA level among hepatocyte cultures in DMSO-treated samples (Figure S2) with gender and age of donors, no correlation was observed. Nevertheless, interindividual differences became also visible after exposure of the primary hepatocytes to visnagin and khellin (1 µM, 10 µM, and 20 µM). Whereas we observed a clear induction of CYP1A1 transcription in any of the four different hepatocyte cultures, the amplitude of the response varied dramatically from donor to donor (Table 1). Therefore, we decided to further investigate the effect of khellin/visnagin exposure on CYP1A1 expression in primary hepatocytes on protein level. As shown in Figure 4, 10 µM and 20 µM of both furanochromones led to a roughly 3- to 6-fold increase in CYP1A1 protein expression after 48 h, which turned out to be statistically significant for all tested concentrations (except the samples treated with 20 µM khellin). In its band intensity, the furanochromone-induced up-regulation was comparable to that reached upon exposure to 1 µM 3MC (Figure 4).

**Table 1:** Influence of visnagin and khellin treatment on CYP1A1 mRNA expression in human primary hepatocytes.

	**CYP1A1 mRNA fold induction**			
	**LH40**	**LH42**	**Hep220586**	**Hep220624**
**UT**	1.0	1.0	1.0	1.0
**VIS 1 µM**	-	7.7	-	10.8
**VIS 10 µM**	63.3	12.0	20.8	28.9
**VIS 20 µM**	43.9	11.1	-	36.1
**KHEL 1 µM**	-	7.5	-	8.8
**KHEL 10 µM**	95.7	34.4	30.0	27.6
**KHEL 20 µM**	60.6	5.0	-	32.6
**3MC 1 µM**	62.8	22.7	206.5	71.1
**TCDD 5 nM**	234.5	38.5	-	-

Human hepatocytes were incubated with visnagin (VIS; 1 µM-20 µM) or khellin (KHEL; 1 µM-20 µM), 1 µM 3MC, 5 nM TCDD, and/or vehicle (DMSO; 0.1% v/v) for 24 h. The data are mean from duplicate measurements and are expressed as fold induction over DMSO-treated cells (UT). The copy numbers were normalized to GAPDH mRNA expression.

The observed increase in CYP1A1 protein expression upon khellin/visnagin exposure raised the idea of elevated catalytic CYP1A activities. Thus, we performed EROD assays of two primary hepatocyte cultures from different donors (LH45, Hep220670). However, in contrast to the applied control substances (3MC and TCDD), which induced EROD activity in both cultures, we only observed a very weak increase in CYP1A enzyme activity upon exposure to 1 µM, 10 µM or 20 µM of either khellin or visnagin (Table S1). Since we observed a significant induction in CYP1A1 protein expression in primary hepatocytes from four different donors, it is highly likely that the tested furanochromones also exhibited CYP1A inhibitory properties, as previously observed in the respective HepG2 experiments (Figure 2E).

In aggregate, we have found that the two furanochromones khellin and visnagin are potent activators of AHR-dependent signaling processes in human primary hepatocytes as well as in a human hepatocarcinoma cell line. Simultaneously, both test compounds are also efficient inhibitors of CYP1A-driven catalyzes, indicating that these compounds may interfere with the metabolic fate of PAHs, drugs, and steroid hormones. The quantitative expression analyses performed in HepG2 cells further indicated that visnagin and khellin modulate the expression of genes whose encoded products (VEGF, PAI-2, AHRR) are involved in regulation of cell growth, differentiation, migration, and apoptosis. At least for khellin, the doses applied in this study are pretty close to those found in human individuals. For instance, it was reported that 2 h to 5 h after ingestion of a single dose of 100 mg khellin, an amount commonly applied during KUVA therapy, peak levels of 4.9 µM to 8.4 µM were reached in the serum of vitiligo patients [55]. Since these levels were achieved after oral uptake, and khellin is rapidly bioavailable, it is tempting to speculate that the liver is initially exposed to even higher khellin concentrations. Thus, the effects observed in our study may indeed occur within the human body. Although the serum levels of khellin reached upon usage of *Ammi* *visnaga* extracts as herbal remedy are quite unknown, the fact that the complete bioavailability of khellin is achieved faster when supplied as component of the whole plant extract than as pure formulation [56], indicates that such applications may also cause significant levels of bioactive khellin in the blood. Accordingly, it cannot be excluded that the high levels of khellin, which are probably present in liver, may be causative for the hepatotoxicity observed in vitiligo patients under systemic KUVA therapy, as indicated by elevated liver transaminases in 7% to 25% of the recipients [55,57]. However, if activation of AHR signaling is involved in khellin-induced hepatotoxicity is not known up-to-now. An interesting issue to elucidate in future studies is to clarify the AHR-activating potential of khellin in melanocytes. Since the AHR was previously shown to mediate UVB-induced skin pigmentation, either by stimulating melanogenesis [27] or melanocyte proliferation [28], khellin-mediated AHR activation may, at least in part, contribute to the re-pigmentation of vitiligo skin under KUVA therapy.

In summary, we have identified khellin and visnagin, two furanochromones probably relevant regarding human exposure, as activators of the AHR in human primary hepatocytes and HepG2 hepatocarcinoma cells. Both compounds increased the expression of several AHR target genes, but simultaneously acted as potent inhibitors of CYP1A monooxygenases. Therefore, we conclude that, especially with regard to the potential health risk for individuals under KUVA therapy, both the putative adverse effects as well as possible drug-drug interactions of khellin and structurally related chemicals have to be carefully elucidated in future toxicological studies to minimize unpredicted side-effects.

## Supporting Information

Figure S1 **The effect of visnagin and khellin exposure on CYP1B1 protein in HepG2 cells.** HepG2 were treated with visnagin (VIS; 20 µM), khellin (KHEL; 20 µM), 5 µM 3MC and/or vehicle (DMSO; 0.1% v/v) for 16 h. Thereafter, western blotting analyses for detection of CYP1B1 and GAPDH were performed as described in Materials and Methods section The representative western blot analysis of two independent experiments (passages) is presented.
(TIF)

Figure S2 **Basal level of CYP1A1 mRNA among hepatocyte cultures.** DMSO-treated human hepatocytes samples (UT) were subjected to PCR analysis as described in Materials and Methods section. The data are mean from triplicate measurements and are expressed as fold induction over DMSO-treated cells (UT) from culture with lowest CYP1A1 basal expression (LH40). The copy numbers were normalized to GAPDH mRNA expression.
(TIF)

Table S1 **Effect of visnagin and khellin exposure on EROD activity in primary hepatocytes.** Two different cultures of human hepatocytes (LH45, Hep220670) were incubated with visnagin (VIS; 1 µM-20 µM), khellin (KHEL; 1 µM-20 µM), 1 µM 3MC, 5 nM TCDD, and vehicle (DMSO; 0.1% v/v) for 48 h. Catalytic enzyme activity was determined from 6 wells/culture as described in the Materials and Methods section. Data are presented as mean ± standard deviation. Statistical significance (*p* ≤ 0.05) was calculated separately for each culture using paired student’s T-test.
(DOC)
