# Supplementary figures and images for: Khellin and Visnagin Differentially Modulate AHR Signaling and Downstream CYP1A Activity in Human Liver Cells
Source: PLoS One. 2013 Sep 19;8(9):e74917. doi: 10.1371/journal.pone.0074917 (PMC3777991; doi:10.1371/journal.pone.0074917)

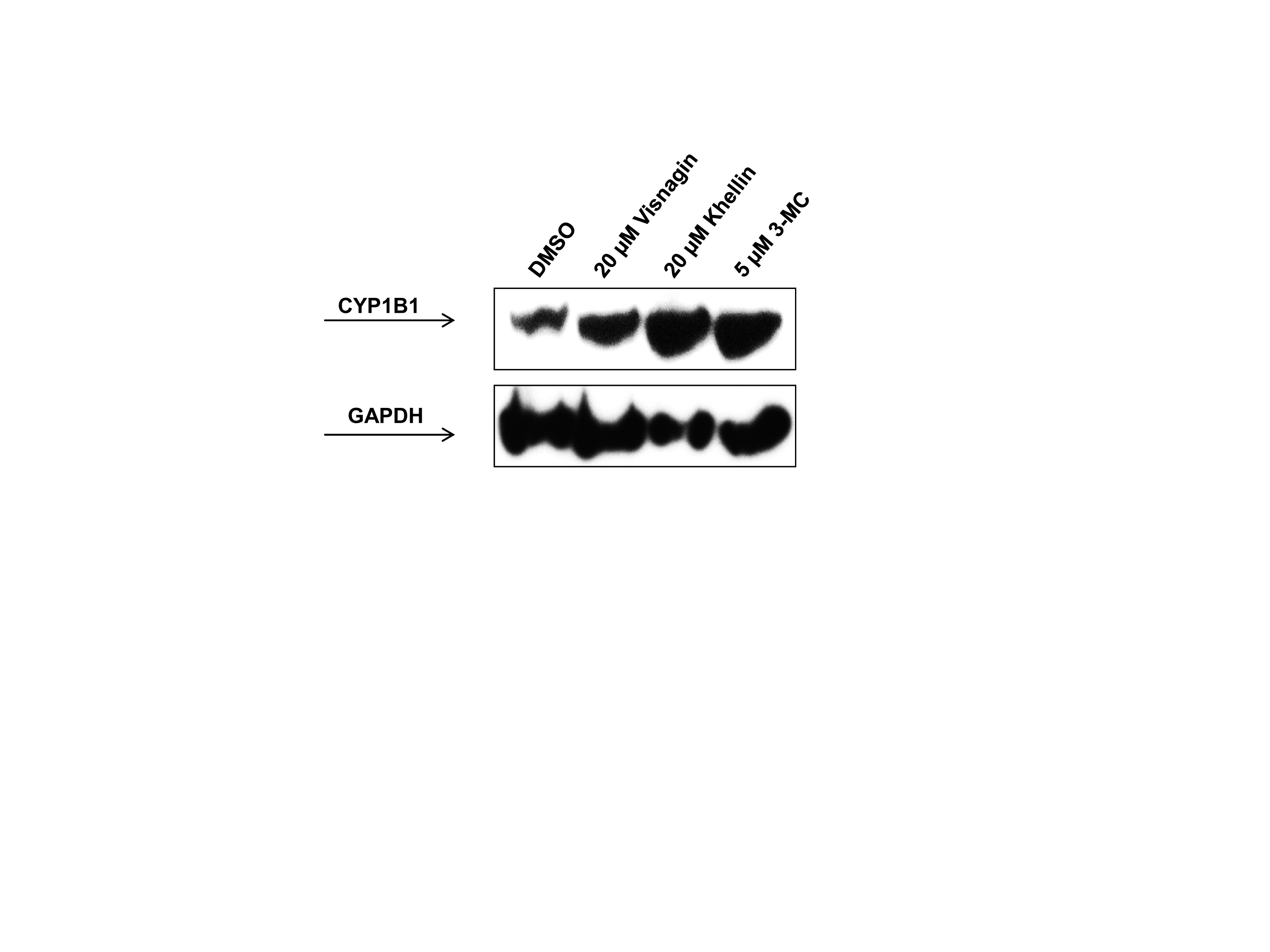

Supplement: Figure S1 — The effect of visnagin and khellin exposure on CYP1B1 protein in HepG2 cells. HepG2 were treated with visnagin (VIS; 20 µM), khellin (KHEL; 20 µM), 5 µM 3MC and/or vehicle (DMSO; 0.1% v/v) for 16 h. Thereafter, western blotting analyses for detection of CYP1B1 and GAPDH were performed as described in Materials and Methods section The representative western blot analysis of two independent experiments (passages) is presented. (TIF) [file pone.0074917.s001.tif]

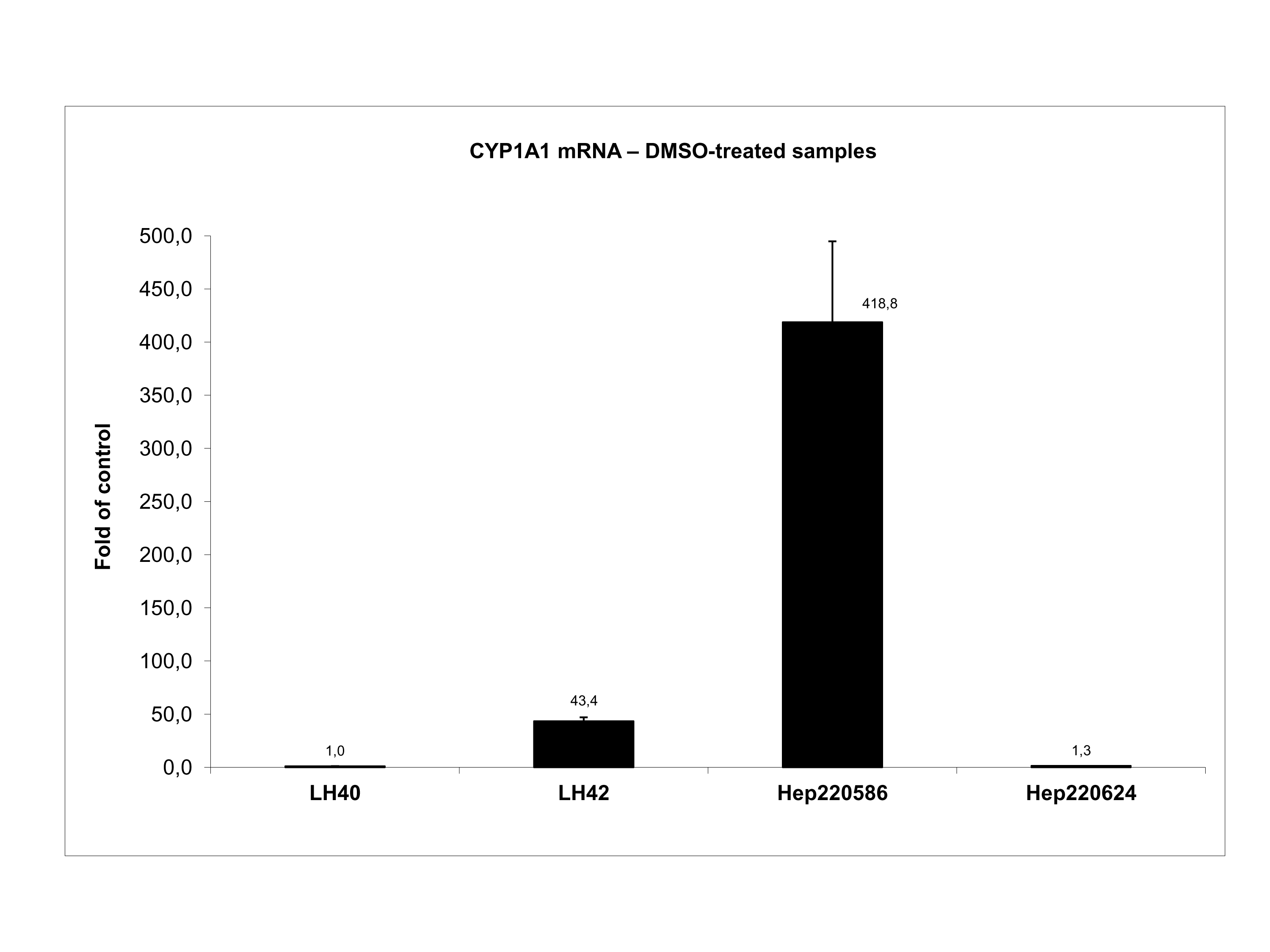

Supplement: Figure S2 — Basal level of CYP1A1 mRNA among hepatocyte cultures. DMSO-treated human hepatocytes samples (UT) were subjected to PCR analysis as described in Materials and Methods section. The data are mean from triplicate measurements and are expressed as fold induction over DMSO-treated cells (UT) from culture with lowest CYP1A1 basal expression (LH40). The copy numbers were normalized to GAPDH mRNA expression. (TIF) [file pone.0074917.s002.tif]
