# Supplementary material for: Khellin and Visnagin Differentially Modulate AHR Signaling and Downstream CYP1A Activity in Human Liver Cells
Source: PLoS One. 2013 Sep 19;8(9):e74917. doi: 10.1371/journal.pone.0074917 (PMC3777991; doi:10.1371/journal.pone.0074917)
Supplement: Table S1 — Effect of visnagin and khellin exposure on EROD activity in primary hepatocytes. Two different cultures of human hepatocytes (LH45, Hep220670) were incubated with visnagin (VIS; 1 µM-20 µM), khellin (KHEL; 1 µM-20 µM), 1 µM 3MC, 5 nM TCDD, and vehicle (DMSO; 0.1% v/v) for 48 h. Catalytic enzyme activity was determined from 6 wells/culture as described in the Materials and Methods section. Data are presented as mean ± standard deviation. Statistical significance (p ≤ 0.05) was calculated separately for each culture using paired student’s T-test. (DOC) [file pone.0074917.s003.doc]

|  | **Fold induction** | |
| --- | --- | --- |
|  | **LH45** | **Hep220670** |
| **DMSO** | **1.00 ± 0,03** | **1.00 ± 0,04** |
| **Vis 1 μM** | **1.00 ± 0,02** | **1.11 ± 0,02*** |
| **Vis 10 μM** | **1.00 ± 0,06** | **1.04 ± 0,02** |
| **Vis 20 μM** | **1.04 ± 0,03*** | **1.03 ± 0,06** |
| **Khel 1 μM** | **1.00 ± 0,06** | **1.17 ± 0,05*** |
| **Khel 10 μM** | **0.98 ± 0,04** | **1.14 ± 0,08*** |
| **Khel 20 μM** | **1.00 ± 0,02** | **1.04 ± 0,03** |
| **3MC 1 μM** | **3.92 ± 0,42*** | **4.60 ± 0,24*** |
| **TCDD 5 nM** | **15.60 ± 0,96*** | **21.10 ± 0,57*** |
